# Premedical Education Experiences of First-Generation College Graduates

**DOI:** 10.1001/jamanetworkopen.2025.49106

**Published:** 2025-12-23

**Authors:** Branden Eggan, Hyacinth R. C. Mason, Devasmita Chakraverty, Jacqueline M. Diaz, Valerie Rivera, Hannah L’Etoile, Brandon Ascencio, Catherine Havemann, Regina G. Russell, Tasha R. Wyatt, Dowin Boatright, Mytien Nguyen

**Affiliations:** 1 Department of Neuroscience and Experimental Therapeutics, Albany Medical College, Albany, New York; 2 Leadership in Medicine Program, Union College, Schenectady, New York; 3 Biology Department, Siena College, Loudonville, New York; 4 Office of Student Affairs, Tufts University School of Medicine, Boston, Massachusetts; 5 Department of Family Medicine, Tufts University School of Medicine, Boston, Massachusetts; 6 Department of Public Health and Community Medicine, Tufts University School of Medicine, Boston, Massachusetts; 7 Ravi J. Matthai Centre for Educational Innovation, Indian Institute of Management Ahmedabad, Ahmedabad, Gujarat, India; 8 Tufts University School of Medicine, Boston, Massachusetts; 9 Health Professions Program, Siena College, Loudonville, New York; 10 Brown University School of Public Health, Providence, Rhode Island; 11 Albany Medical College, Albany, New York; 12 Department of Emergency Medicine, University of North Carolina at Chapel Hill, Chapel Hill; 13 Office for Undergraduate Medical Education, Vanderbilt University School of Nursing, Nashville, Tennessee; 14 Center for Health Professions Education, Uniformed Services University, Bethesda, Maryland; 15 Ronald O. Perelman Department of Emergency Medicine, New York University Grossman School of Medicine, New York, New York; 16 Department of Immunology, Yale School of Medicine, New Haven, Connecticut

## Abstract

**Importance:**

First-generation undergraduate students are underrepresented in medicine and face challenges that affect their application and matriculation to medical school.

**Objective:**

To understand the barriers and available support at the individual, interpersonal, and organizational levels for first-generation students in premedical programs.

**Design, Setting, and Participants:**

This qualitative study was part of a larger project on professional identity formation among US medical students from low socioeconomic backgrounds with a focus on first-generation undergraduate students. Semistructured online interviews of 48 students (37 of whom were first-generation students) from 27 US medical schools were conducted from November 1, 2021, through April 30, 2022, and secondary analyses regarding their premedical experiences were performed. Interviews were recorded, transcribed, deidentified, assigned an alphanumeric code, and analyzed using an inductive thematic approach from June 1 through December 30, 2024. Participants were offered a $20 gift card in appreciation for their time.

**Exposure:**

Experiences of students identifying as first-generation college students in premedical training.

**Main Outcomes and Measures:**

Main outcomes included themes and subthemes at the individual, interpersonal, and organizational levels constructed from interview data. Bronfenbrenner’s ecological systems theory was used as a guiding interpretive framework to analyze participants’ experiences within their broader social and environmental contexts. This model allowed examination of how individual perspectives were shaped by interactions across personal, social, and structural contexts. The team engaged in reflexive discussions, memos, and consensus-based refinement to identify key themes.

**Results:**

Among 37 participants identified as first-generation students, 21 (56.8%) identified as female (mean [SD] age, 27.3 [2.8] years). One participant (2.7%) identified as American Indian or Alaska Native; 7 (18.9%) as Hispanic, Latino, or Spanish origin; 8 (21.6%) as non-Hispanic Asian or Asian American; 9 (24.3%) as non-Hispanic Black or African American; and 12 (32.4%) as non-Hispanic White. Analyses revealed key themes across all 3 levels of the ecological systems model: individual, interpersonal, and organizational. Individual themes included personal and family background, such as financial instability and lack of clinical exposure. Interpersonal themes included access to premedical advisors, faculty mentors, and peer networks. Organizational themes included limited institutional resources and pathway programs.

**Conclusions and Relevance:**

In this qualitative study of first-generation students, financial instability, limited medical school–related social connections and resources, and unfamiliarity with academic systems were found to uniquely burden these students. By identifying and improving resources such as tailored mentorship, individualized advising, and participation in pathway programs, medical schools can help reduce structural barriers for aspiring physicians who are first-generation college students.

## Key Points

**Question:**

How does first-generation college status shape the premedical experiences of medical students at the individual, interpersonal, and organizational levels, and what strategies can improve support across these domains?

**Findings:**

In this qualitative study of 37 medical students who were first-generation college students, participants reported facing many premedical challenges, including financial instability and limited medical school–related social connections. Individual themes identified included financial instability and lack of clinical exposure; interpersonal themes included access to premedical advisors, faculty mentors, and peer networks; and organizational themes included limited institutional resources and pathway programs.

**Meaning:**

These findings suggest that interpersonal and organizational support can help address obstacles faced by first-generation students during their premedical education.

## Teaser

This qualitative study explores the individual, interpersonal, and organizational barriers experienced by first-generation undergraduate students in premedical training.

## Introduction

First-generation college students constitute 54% of undergraduates nationwide,^1^ but in the 2024-2025 academic year, they accounted for just 13.8% and 11.0% of medical school applicants and matriculants, respectively.^2^ Data consistently show that a greater percentage of first-generation undergraduate students are from racial and ethnic minority backgrounds compared with their continued-generation peers.^3^ Research has shown that medical student and health care team diversity is correlated with improved educational experiences, better patient care outcomes, and more culturally competent health care.^3,4^

With most physicians and medical students coming from high socioeconomic backgrounds, bringing students from diverse socioeconomic backgrounds into medicine is crucial to improving patient care. After the US Supreme Court issued a ruling ending race-conscious admissions in 2023,^5^ the Association of American Medical Colleges (AAMC) clarified that the Supreme Court decision “was not a condemnation of diversity or a school’s goals related to the educational benefits associated with exposure to a broad array of perspectives,” stressing that these goals are “plainly worthy.”^6^

Disparities between students of different socioeconomic backgrounds begin long before medical school matriculation. While a growing body of research exists on the medical school experiences of first-generation students,^3,4,7,8^ less is known about their premedical experiences.^9,10^ Students without strong connections to the medical profession are less likely to stay on the premedical path^11,12,13^ or apply to and matriculate into medical school.^2,4,14^ In this study, we explored how premedical experiences at the individual, interpersonal, and organizational levels are shaped by first-generation college student status.

## Methods

### Study Design

This qualitative study was part of a larger mixed-methods study exploring the professional identity formation of US-based medical students from backgrounds of lower socioeconomic status, including students who identified as first-generation college graduates or were from low-income backgrounds. Participants self-reported their parental educational level, family income, sex, age, and race and ethnicity (including American Indian or Alaska Native, Hispanic, non-Hispanic Asian or Asian American, non-Hispanic Black or African American, and non-Hispanic White). Following Institutional Review Board approval from Yale University, we conducted semistructured interviews on an online platform (Zoom; Zoom Communications Inc) with participants between November 1, 2021, and April 30, 2022, discussing their premedical experiences. Participants received a $20 gift card for their time. The interviews were recorded with participant consent, transcribed, deidentified, and assigned an alphanumeric code. We analyzed data using an inductive thematic approach,^15^ adhering to the Standards for Reporting Qualitative Research (SRQR) guidelines.^16^

### Research Team

The coding team included 3 health profession researchers (B.E., H.R.C.M., and D.C.), 2 medical students (J.M.D. and B.A.), and an undergraduate prehealth advisor (V.R.). Each team member had academic expertise in medical education, lived experience as a scholar from a first-generation background, or both. Each transcript was coded by at least 1 trainee and 1 nontrainee. The team discussed member positionality and reflexivity to examine how individual perspectives can influence analysis and interpretation. Through memos and regular coding meetings, we shared reflections, challenged assumptions, and refined interpretations, deepening our understanding of participants’ premedical experiences.^17^

In this exploratory study, we used Bronfenbrenner’s ecological systems theory^18^ as a guiding interpretive framework to analyze participants’ experiences within their broader social and environmental contexts during their premedical years. This theoretical model conceptualizes human development as occurring within a set of nested, interrelated systems that influence individuals in both direct and indirect ways. It includes the individual (eg, family), the interpersonal (eg, peer and mentor networks), and the organizational (external settings that indirectly impact the student, such as the medical school) systems.

### Trustworthiness

We used consensus coding to improve reliability and trustworthiness of the analysis. Our research team had varied backgrounds and experiences; this diversity of perspectives strengthened our analysis. Our findings were validated through member checks with 2 participants via email. Both confirmed that the findings aligned with their experiences and offered refinements, which were incorporated.^19^

### Data Analysis

The study team analyzed data between June 1 and December 30, 2024. We used the Dedoose platform, version 9.0.17 (Sociocultural Research Consultants LLC), to conduct independent line-by-line coding of 5 randomly chosen transcripts, and then refined and consolidated codes through group discussion and consensus. We analyzed and coded the remaining transcripts independently using inductive thematic analysis and constant comparison, resolving discrepancies through consensus. The team constructed themes, created an audit trail through memos, and documented our assumptions and biases throughout and after coding.^17,20^ Data saturation was achieved when themes became repetitive and no new information emerged from successive transcripts.

## Results

### Demographics

Participants were recruited through specialized US-based medical listservs for students from first-generation backgrounds. Of the 116 medical students who expressed interest in participation, 67 met the eligibility criteria (ie, self-reported that neither a parent nor a guardian had attained a bachelor’s-level college degree). A total of 48 medical students were interviewed, and of these, 37 self-identified as first-generation college students. Among 37 participants, 21 (56.8%) identified as female and 16 (43.2%) as male, with a mean (SD) age of 27.3 (2.8) years. One participant (2.7%) identified as American Indian or Alaska Native; 7 (18.9%), Hispanic, Latino, or Spanish origin; 8 (21.6%), non-Hispanic Asian or Asian American; 9 (24.3%), non-Hispanic Black or African American; and 12 (32.4%), non-Hispanic White (Table 1).

**Table 1.** Participant Demographic Characteristics

Characteristic	No. (%) (N = 37)
Age, mean (SD), y	27.3 (2.8)
Age group, y	
23-25	13 (35.1)
26-29	15 (40.5)
≥30	9 (24.3)
Sex	
Female	21 (56.8)
Male	16 (43.2)
Race and ethnicity	
American Indian or Alaska Native	1 (2.7)
Hispanic, Latino, or Spanish origin	7 (18.9)
Non-Hispanic Asian or Asian American	8 (21.6)
Non-Hispanic Black or African American	9 (24.3)
Non-Hispanic White	12 (32.4)
Highest level of education completed by parents or guardians	
Elementary	4 (10.8)
Some high school	1 (2.7)
High school graduate or GED	23 (62.2)
Associate degree or equivalent	4 (10.8)
Some college, no bachelor’s degree	4 (10.8)
NA	1 (2.7)
Medical school program type	
MD	35 (94.6)
DO	2 (5.4)
Phase of school	
Clinical	23 (62.2)
Preclinical	14 (37.8)
Family income	
$0-$49 999	25 (67.6)
$50 000-$119 999	10 (27.0)
$120 000-$199 999	1 (2.7)
Do not know	1 (2.7)
SNAP or federal assistance programs growing up	
Yes	18 (48.6)
No	15 (40.5)
Do not know	3 (8.1)
Prefer not to answer	1 (2.7)

Abbreviations: DO, doctor of osteopathic medicine; GED, General Educational Development; MD, doctor of medicine; NA, not available; SNAP, Supplemental Nutrition Assistance Program.

### Major Themes

The premedical experiences of study participants were analyzed using an adapted version of Bronfenbrenner’s ecological systems theory as an interpretive framework.^19^ Themes are presented across 3 interconnected levels: individual (personal and family background), interpersonal (faculty advisors or mentors, peers, and others with whom the participants interacted directly), and organizational (institutions and programs).

#### Individual-Level Themes

##### Individual and Family Health-Related Experiences

Family background played a significant role in shaping participants’ journeys, including the reasons they considered a medical career. One student shared that growing up, they felt as if “I can’t tell my mom that I’m sick because I don’t know if she can afford medicine.… That made me pursue medicine because I think that I could change some of that” (participant 7).

Similarly, another student shared that their reasons for pursuing medicine were based on their family’s lack of access to quality health care and their experience serving as an interpreter in the health care setting: “A lot of times, the doctor was like, ‘Do they understand?’ and I’m like, ‘Okay, now I’m translating.’ … I’m seeing the barriers and realize, ‘Oh, there’s no physicians that look like me,’ or ‘Why aren’t there physicians who speak our language?’ That stuck with me” (participant 29).

##### Navigating Without a Map

Many students struggled to conceptualize the medical profession without physician role models. One student stated that while they had aspired to become a physician since childhood, “I thought that was so above me just because I had no exposure to people who were physicians” (participant 42).

Feelings of invisibility, not belonging, and imposter syndrome were common during the premedical phase. Participants shared being expected to feel “very grateful to get accepted to this wonderful college as a first-generation [student], and just figure it out” (participant 40). Another student shared that even when they were successfully navigating the premedical path, there was a sense that “people are going to figure out that I don’t belong…. They’re going to realize that, and then just that’s it, game over for me” (participant 47).

Participants also struggled with a lack of knowledge about medical education and having “to go above and beyond what others did to find the resources … and information I needed to succeed … and become a better advocate for myself” (participant 43). Many participants shared that it was often unclear what was expected: “There is such a huge hidden curriculum … not only as it relates to surviving undergrad but going through the admissions process for med school” (participant 22) and “When you’re first-generation, you just don’t know how to succeed at the game sometimes … every aspect seems to have a strategy, and I’m starting from scratch” (participant 7).

##### Financial Barriers

First-generation college students’ premedical educational trajectories were often impacted by financial burdens as they balanced education and employment. Many felt that faculty, staff, and other institutional leaders who were not first-generation college students were not aware of the financial burdens these applicants experienced, and the potential consequences: “I struggled academically my last year and almost dropped out just because it was too much to manage. I was working 2 jobs and trying to go to school. I was living out of my car. It was a heavy year. I didn’t know what to do or who would understand” (participant 9).

Fulfilling admission requirements such as obtaining clinical experience and taking the Medical College Admissions Test (MCAT) added financial strain and sometimes was a deterrent from pursuing medicine. Unlike their continuing-generation peers, many first-generation students could not afford MCAT tutors and felt underprepared: “I didn’t pull on the same resources others had because I didn’t have the extra $1000 here, $400 there to do that. The MCAT prep courses; they’re like $4000 to $5000 now, which is absurd” (participant 22); “My family can never afford this. So why am I even going to try? Even if you have the brains or whatever, the skill set to get in, people don’t want to try because it’s so expensive” (participant 2); and “Clinical exposure was one of the weakest points in my application because I was working instead. I bartended, dog walked, nannied, [and] made and sold clothes to make ends meet” (participant 45).

Many participants had to take loans, work additional jobs, and save for years to be able to afford these opportunities, something that drained their time, energy, and well-being: “[I] saved for 3 years just to afford applications and traveling to interviews” (participant 2). Students sometimes reached out to peers for financial support: “I took a loan at one point from a friend who was on scholarship, and I was like ‘Hey, this is for med school, I swear—I need to apply to more schools if I’m going to be able to do this’” (participant 22).

Financial constraints also affected participants’ opportunities in other ways. As one student shared, the cost of traveling for interviews limited the schools they applied to: “I only applied to schools that were within driving distance because I couldn’t afford airfare” (participant 30).

Students reported spending significant time navigating the basics of financing a premedical education: “Instead of spending a couple of months figuring out what to do, they spend that time just doing, right off the bat, what undergrads or med schools or programs are looking for. It gives a leg up” (participant 36) and “I didn’t have the resources to take time off to just study for the MCAT. Like a lot of people, I didn’t have that luxury. I need to either have a job or go to school” (participant 15).

#### Interpersonal-Level Themes

##### Premedical Advisors and Mentors

Mentoring and support were pivotal in helping first-generation students mitigate challenges throughout their premedical journey. One student noted, “There were people that knew other people, they knew how to navigate the system, and that was what I needed” (participant 29). Another noted that “because I was … part of a [pathway] program at my undergraduate school … I had mentors who knew what I was going through, and who would just keep it real and honest with me” (participant 5).

However, some mentors did not always seem to understand students’ challenges: “I remember wanting to do a [research] project but being unsure where to start…. I remember meeting with one of my advisors. They said, ‘Oh, you just do it. Just think about a project, figure out where you want to go next.’ But what’s difficult is that if you don’t know where to start or if you don’t know what questions to even ask because you don’t know what you’re supposed to be doing, you get nowhere” (participant 2).

##### Peer Networks

Many relied on trial and error and informal sources of information, such as peers, to fill gaps in formal information about medical education. Many students belonged to first-generation student organizations such as the National First-Generation and Low-Income in Medicine Association (FGLIMed)^21^ and emphasized the importance of affinity groups during the premedical years: “I know people who dropped out of undergrad simply because they didn’t feel like they belonged there or felt seen. The group [FGLIMed] gave me the sense of ‘I deserve to be here.’ I cannot understate the importance of that experience in undergrad. That gave me a lot more confidence” (participant 30).

Peer support was particularly important when students felt isolated: “My friends helped me feel like I wasn’t crazy because the challenges I faced weren’t what most students experience” (participant 2). Additionally, participants made up for the information they lacked by observing and imitating peers: “I found people who were pre-med and essentially copied them” (participant 45).

##### Connections and Social Capital

Support from social connections was valuable in navigating processes unfamiliar to first-generation students. “My friend’s mom helped me apply for [college] scholarships. [They] sat down with me and was like, ‘Let’s apply for scholarships. Let’s call schools. Schools have money. We’ll negotiate.’ The things that my parents didn’t know about and made a difference” (participant 26). Lack of social connections was a source of frustration, creating hurdles to acquiring clinical exposure and research experience: “Not having family members anywhere near the medical field, I had a hard time shadowing, getting in the door for a lot of important experiences, or even knowing certain jobs existed that would give me exposure” (participant 26).

#### Organizational-Level Themes

##### Lack of Formal Information About Applying to Medical School

Participants lacked an understanding of what they could do to strengthen their application. Many shared that continuing-generation students had an implicit or tacit understanding that they lacked about topics such as coursework, extracurricular experience, and the MCAT. One participant described the medical school application process as “a mess. I had no idea what I was doing…. I felt so out of it, trying to hide what I didn’t know. What’s the MCAT? Do I need to shadow? What courses should I take? It was embarrassing to ask because everyone else seemed to know” (participant 30). Another student said, “It was this trial-and-error experience. Trying to get good grades and extracurriculars and hoping I’d know how to navigate it. I saw other students engaged in activities, ask for research, commit to extracurricular opportunities, and apply way ahead for summer opportunities. I just felt like everybody else was ahead of me and I did not know anything, while everybody else knew” (participant 43).

##### Benefits of Pathway Programs

Pathway and preparatory programs in high school and college were pivotal in promoting interest in medicine: “With guidance from [pathway program] I was able to get more financial aid from school that lifted such a huge stress off me and helped me get into a better [undergraduate] school for premed than I would have tried for” (participant 3). Another described the premedical experience as “an expensive journey … the exams are expensive … traveling to interviews … applications that you’re spending money for…. The [AAMC] Fee Assistance Program and my [pathway program] helped me a lot…. That allowed me to apply to more schools than I would have if I didn’t have that funding” (participant 29).

Beyond financial help, pathway programs also provided emotional support and validation. One student reflected that “Having that infrastructure of people for years telling me like, ‘Yeah, this is going to be different for you than other people, you deserve to be there just as much as anyone,’ was really helpful” (participant 30).

## Discussion

In this qualitative study, we found that first-generation premedical students face challenges on individual, interpersonal, and organizational levels. Study participants shared that financial instability, lack of premedical program–related social connections and capital, and unfamiliarity with the requirements for medical school admission were deterrents to entering medicine. For many first-generation students, overcoming these barriers was an isolating journey with self-doubt and imposter syndrome, a finding in line with existing research showing that first-generation students across all stages of medical training, despite strong academic records, often feel like frauds and struggle to internalize their success.^22^

While existing research describes significant challenges encountered by first-generation undergraduates^3^ and medical school students,^7,8,23,24^ it is limited on the unique challenges of first-generation premedical undergraduate students. Financial barriers emerged as a prominent theme in this study, with many students struggling to afford costs associated with the premedical path. Nationally, only 6.1% of medical students are from the bottom 2 income quartiles,^25^ highlighting the deep socioeconomic disparities in access to medical education. First-generation students are less likely to matriculate into both MD and MD-PhD programs compared with their continuing-generation student peers.^4,26^ However, a result of financial strain not noted in existing literature is an increased time to complete undergraduate degrees. Some extended their premedical education to both allow more time for preparation and to earn money to support themselves and pay for the admissions process. This contributed to heightened stress and poor well-being as well as lengthening the path to medicine. Of the 37 first-generation students in this study, 9 (24.3%) were 30 years or older, indicating that some may have faced significant delays in beginning their medical training.

Furthermore, a limited understanding of the medical pathway requires some first-generation students to navigate this path through trial and error, potentially increasing their overall financial investment. Organizational issues such as the high cost of study materials, application and interview expenses, and a reliance on social capital to effectively navigate the process seem to disproportionately burden first-generation undergraduate students. This is in line with a 2024 study by Drake^27^ showing that first-generation undergraduate students with low income face heightened risks of academic underperformance and attrition due to the combined effects of limited financial resources, lack of college knowledge, and reduced access to support networks.

The challenges of premedical education were mitigated by mentorship, peer networks, social connections, and pathway programs. In our study, participants shared that pathway programs that provided financial and interpersonal support were pivotal in retention of students in medicine. This finding is in line with other research on the benefits of pathway programs in providing structured academic support, mentoring, and experiential opportunities for students. Despite these challenges during premedical years, research shows that first-generation students are just as likely as their peers to be inducted into the Alpha Omega Alpha national medical honor society, underscoring their ability to thrive when given adequate support.^28^

In addition to pathways programs, building strong relationships between prehealth advisors and students can ease transitions by providing guidance on applications, financial aid, and academics. Advisors can also connect students with role models in the field, fostering a sense of belonging and confidence. Based on our findings, we provide a detailed list of questions and suggestions educators and administrators can use to evaluate their support for first-generation premedical students in Table 2. Strong support systems from undergraduate institutions, medical schools, and other entities, such as the Joint Admission Medical Program^29^ or the Summer Health Professions Education Program,^30^ can help unlock opportunity for all students regardless of background and create a health care workforce better equipped to meet the needs of all patients.

**Table 2.** Questions and Recommendations to Enhance First-Generation Premedical Students’ Medical Career Opportunities

Bronfenbrenner’s ecological system level	Key questions for medical educators and institutions	Recommendations
Individual	Personal barriers to medical school	Expand support for personal challenges
	How can we foster relationships among first-generation students, peers, and mentors? Do first-generation students have equitable access to extracurricular and clinical experiences? Are academic and financial resources transparent and accessible? Does the institution regularly host workshops that enable first-generation students to build community?	Build support networks by establishing structured peer and faculty mentorship programs Transparent clinical exposure: Provide paid or low-cost clinical opportunities and develop relationships with clinicians Financial equity: Proactively inform students of all available fee assistance programs Organize regular workshops that include peer socializing and host discussions on first-generation strengths and advising
Interpersonal	Premedical advising and peer networks	Improve advising
	Are premedical advisors trained to effectively support first-generation students? How can advising be more proactive and structured to help first-generation students succeed? How do students obtain reliable and equity-minded information about applying to medical school? Do students know about available academic and financial resources, and are they truly accessible to all? Are we actively working to illuminate hidden curriculum disparities? How do we help first-generation students build peer and faculty networks? How can we help first-generation students obtain more equitable access to shadowing health care professionals?	Implement mandatory first-generation–focused premed advisor training Structured advising: Require proactive, structured advising check-ins Provide structured workshops on premedical requirements, time management, and resilience-building strategies Accessible resource hub: Develop a centralized website or application with first-generation–specific premedical guidance Address the hidden curriculum: Promote workshops through student groups and in courses focused on topics such as biomedical ethics and health policy Expand mentorship by creating near-peer mentorship programs Facilitate early exposure to health care careers by creating formal, school-based shadowing programs, premed boot camps, and affinity groups
Organizational	Formal information on applying to medical school	Provide transparent and accessible admissions information
	Is admissions information clear, accessible, and equitable for all applicants? Do admissions committees recognize applicants’ achievements in the context of systemic barriers? Are students aware of and supported in accessing fee assistance programs in a timely manner?	Transparent admissions guidance: Create a standardized, user-friendly medical school admissions guide with clear timelines, FAQs, and first-generation–specific support Holistic review: Train admissions committees to recognize systemic barriers and value resilience and lived experience in applicants Support for application costs: Establish institutional funds or alumni-sponsored scholarships for Medical College Admissions Test preparation, interview travel, and application fees
	Pathway programs	Expand inclusive pathway programs
	Are pathway programs reaching students who need them most to meet our school’s mission? Do students have access to bridge programs or postbaccalaureate options if they need additional preparation?	Partnerships and affiliations: Strengthen partnerships with community colleges, tribal colleges, Historically Black Colleges and Universities, and Hispanic-Serving Institutions and collaborate with youth-supporting private entities Expand pathways: Offer flexible premedical pathways (eg, part-time coursework, structured glide years) and expand funded postbaccalaureate and structured gap year programs
	Limited resources and longer trajectories	Redefine premed success to reflect equity
	How do we support students who lack financial flexibility for research years or extra coursework? Are we acknowledging and accommodating different premedical timelines (eg, gap years, community college transfers)? Are we advocating for funding for our students who need additional financial support to cover essential premedical expenses? Are we regularly maintaining and analyzing anonymized student outcome data to understand the reality of the premedical pathway for first-generation students? Are merit-based scholarships equitable and inclusive of “distance traveled” as a measure of achievement? How can institutions foster belonging and inclusion in the premed pathway?	Improve access and financial support Remove barriers to enrolling in the AAMC’s Fee Assistance Program Expand institutional funding to cover essential premedical expenses, including clinical and research opportunities Expand need-based financial aid Ensure that scholarships consider achievement in the context of adversity, rather than privileging traditional pathways Connect students with role models who have overcome barriers Highlight success stories through speaker series, mentorship, and media features Culture shift Normalize nontraditional premedical paths Promote longer undergraduate timelines, gap years, and community college pathways as valid premedical trajectories Data-driven premedical advising Maintain anonymized first-generation student outcome data to offer equity-focused premedical guidance

Abbreviations: AAMC, Association of American Medical Colleges; FAQ, frequently asked question.

### Limitations

This study has several limitations. First, the use of convenience sampling through listservs and social media may have introduced selection bias, since all participants self-selected into the study. This may have excluded prospective participants outside these networks, limiting the representativeness of the sample. Additionally, as with all qualitative research, findings are context dependent and not intended to be statistically generalizable. Our sample size (37 participants) is appropriate for an in-depth qualitative inquiry but may not reflect the full diversity of experiences across all institutional or geographic contexts.

Second, the experiences captured reflect those of matriculants who successfully applied to medical school, with a potential bias for optimistic or resilient responses. First-generation students are less likely to be accepted into medical school than their peers, suggesting that these students face significant barriers to admissions to medical school.^4,30,31^ However, the obstacles reported by these successful applicants highlight how pervasive and significant these challenges are. Future research should seek to include the perspectives of first-generation students who pursued premedical coursework but did not apply to or gain acceptance into medical school, as their experiences may offer critical insights into points of attrition and structural inequities.

Third, we did not include a comparison group, such as students from low-income backgrounds who are not first-generation college graduates, or those who were the first in their family to pursue a career in health care but whose parents attended college. As a result, we are unable to determine whether the themes identified are unique to first-generation students or shared more broadly among other groups with limited social, cultural, or economic capital. Future studies using comparative designs are necessary to explore how first-generation undergraduate status intersects with or differs from other axes of marginalization.

Last, it is important to acknowledge that the experiences shared in this study reflect the premedical journeys of students prior to the 2023 US Supreme Court^5^ decision that ended race-conscious admissions policies and current federal efforts to ban diversity, equity, and inclusion initiatives. Future research should use diverse methods to explore whether and how these policies have affected the premedical pathways for first-generation students.

## Conclusions

This qualitative study examining the educational experiences of first-generation premedical students highlights the personal and systemic barriers faced by this population. Our findings emphasize the need for comprehensive, tailored resources to provide equitable educational opportunities for first-generation aspiring physicians.

## Notes

Supplement.
Data Sharing Statement
