# Supplementary material for: Premedical Education Experiences of First-Generation College Graduates
Source: JAMA Netw Open. 2025 Dec 23;8(12):e2549106. doi: 10.1001/jamanetworkopen.2025.49106 (PMC12728652; doi:10.1001/jamanetworkopen.2025.49106)
Supplement: Supplement. — Data Sharing Statement [file jamanetwopen-e2549106-s001.pdf]

Data Sharing Statement

Eggan. Premedical Education Experiences of First-Generation College Graduates. *JAMA Netw Open*. Published December 23, 2025. doi:10.1001/jamanetworkopen.2025.49106

Data
Data available: No
